# A Novel Analytical Framework for Dissecting the Genetic Architecture of Behavioral Symptoms in Neuropsychiatric Disorders

**DOI:** 10.1371/journal.pone.0009714

**Published:** 2010-03-16

**Authors:** Anthony J. Deo, Ramiro Costa, Lynn E. DeLisi, Rob DeSalle, Fatemeh Haghighi

**Affiliations:** 1 Department of Biology, New York University, New York, New York, United States of America; 2 Center for Comparative Genomics and Department of Invertebrate Zoology, American Museum of Natural History, New York, New York, United States of America; 3 Department of Psychiatry, College of Physicians and Surgeons, Columbia University, New York, New York, United States of America; 4 Division of Molecular Imaging and Neuropathology, The New York State Psychiatric Institute, New York, New York, United States of America; 5 Harvard Medical School, VA Boston Healthcare System, Brockton, Massachusetts, United States of America; The University of Hong Kong, China

## Abstract

**Background:**

For diagnosis of neuropsychiatric disorders, a categorical classification system is often utilized as a simple way for conceptualizing an often complex clinical picture. This approach provides an unsatisfactory model of mental illness, since in practice patients do not conform to these prototypical diagnostic categories. Family studies show notable familial co-aggregation between schizophrenia and bipolar illness and between schizoaffective disorders and both bipolar disorder and schizophrenia, revealing that mental illness does not conform to such categorical models and is likely to follow a continuum encompassing a spectrum of behavioral symptoms.

**Results and Methodology:**

We introduce an analytic framework to dissect the phenotypic heterogeneity present in complex psychiatric disorders based on the conceptual paradigm of a continuum of psychosis. The approach identifies subgroups of behavioral symptoms that are likely to be phenotypically and genetically homogenous. We have evaluated this approach through analysis of simulated data with simulated behavioral traits and predisposing genetic factors. We also apply this approach to a psychiatric dataset of a genome scan for schizophrenia for which extensive behavioral information was collected for each individual patient and their families. With this approach, we identified significant evidence for linkage among depressed individuals with two distinct symptom profiles, that is individuals with sleep disturbance symptoms with linkage on chromosome 2q13 and also a mutually exclusive group of individuals with symptoms of concentration problems with linkage on chromosome 2q35. In addition we identified a subset of individuals with schizophrenia defined by language disturbances with linkage to chromosome 2p25.1 and a group of patients with a phenotype intermediate between those of schizophrenia and schizoaffective disorder with linkage to chromosome 2p21.

**Conclusions:**

The findings presented are novel and demonstrate the efficacy of this approach in detection of genes underlying such complex human disorders as schizophrenia and depression.

## Introduction

Emil Kraepelin's descriptions of psychiatric diagnoses at the turn of the 20th century were groundbreaking and remain influential to this day. Kraepelin's dichotomous classification of manic-depressive insanity (bipolar disorder) and dementia praecox (schizophrenia) provides a simple way for conceptualizing an often complex clinical picture and has been extended to include a categorical classification system utilized for a vast array of psychiatric illnesses [1]. The validity of such a categorical classification system has been challenged, as providing an unsatisfactory model of mental illness [2], [3], [4], [5]. In clinical practice, many patients with psychiatric illness do not conform to a prototypical diagnostic category. Findings emerging from many psychiatric research areas and in particular psychiatric genetics are not consistent with the traditional categorical model. For example, family studies reveal notable familial co-aggregation between schizophrenia and bipolar illness and between schizoaffective disorders and both bipolar disorder and schizophrenia [6], [7], [8]. This points to the arbitrary nature of diagnostic boundaries that do not reflect the underlying pathology. Further, linkage and association studies demonstrate shared genetic susceptibility in schizophrenia and bipolar disorder. This has been shown through systematic whole-genome linkage analyses that have identified linkage to common chromosomal regions [6], [7], as well as candidate gene studies whose variants were shown to be associated with both schizophrenia and bipolar disorder [6].

Additionally, the categorical diagnostic model does not adequately accommodate atypical or sub-clinical cases[5]. For example, cases with a mixture of psychotic and affective symptoms are not clearly assigned to the categories of schizophrenia, bipolar disorder or major depression in research into treatment and pathogenesis. Patients with schizoaffective illness represent such cases. Indeed, to date only a single genetic linkage study has been conducted on such a common disorder, and the findings support the potential existence of specific susceptibility loci to psychosis with features of both schizophrenia and bipolar disorder [9]. Compelling evidence is also observed from association studies of cases with a mixture of features from the categorical prototypes that likely constitute distinct yet potentially more homogeneous disease entities. Of note are two candidate disease gene studies. The Neuregulin 1 gene was first reported in studies of schizophrenia within the Icelandic population [10], which also has a risk haplotype that was found to confer the greatest risk in bipolar disorder with mood-incongruent psychotic features and schizophrenia with mania. This haplotype was found to have little effect in cases without both mania and mood-incongruent psychotic features[11]. Also, variations in the D-amino acid oxidase activator gene have been reported to potentially increase susceptibility to episodes of mood disorders in patients suffering from both bipolar disorder and schizophrenia[11].

The limitations of the existing categorical model with arbitrary diagnostic boundaries and hierarchical diagnostic definitions that do not allow for presence of the spectrum of sub-clinical symptoms have impeded progress in psychiatric genetic research. An alternative model is the suggestion that psychiatric disorders are related as part of a continuum of psychosis [3]. A continuum would represent patients with exclusively psychotic and affective symptoms at the extreme ends of the spectrum and those with a mixture of these symptoms (as with schizoaffective disorder) intermediate along this spectrum. In this view, psychiatric disorders represent the extreme variants of personality traits in the general population with “normal” including those who do not meet standards for a medically relevant diagnosis, though do possess symptoms of these disorders [12]. As such, direct examination of the individual's behavioral symptoms without regard to diagnostic category might be more informative for determining the relationship of disease states in different patients and the identification of genetic factors contributing to these behavioral symptoms. The search for such genetic factors must be conducted at multiple phenotypic levels to allow for the possibility of both local effects (affecting a subset of patients) and global effects (affecting the majority of patients with the disorder).

We present an analytical framework for detection of genetic factors contributing to specific subsets of behavioral symptoms. This approach is a paradigm shift from traditional genetic analytic methods, where the genetic analyses are performed within given pre-defined diagnostic categories. In contrast, in this approach, individuals are grouped into a hierarchical network based on shared behavioral symptoms without regard to the diagnostic category to which they had originally been assigned. This form of behavioral clustering is highly flexible allowing for both separation and overlap of clinical symptoms within diagnostic categories. Genetic linkage and association tests can then be preformed using the individual groupings from clustering of individuals with an observed set of symptom profiles. In this way, the genetic determinants underlying a specific cluster of symptoms that define a clinical sub-phenotype may be detected. This framework was especially developed for analysis of data from genetic studies of neuropsychiatric disorders with a wide spectrum of clinical symptoms. With this in mind, we applied this method to analysis of schizophrenia data, where we demonstrate the efficacy of this approach in refining the findings from a previous schizophrenia genome scan [13]. As a proof of principle, we also used a published simulated dataset from the Genetic Analysis Workshop 14 [14] that was designed to model the genetic influences on a complex psychiatric disorder.

## Materials and Methods

### Ethics Statement

The recruitment and diagnosis of patients and their family members are described elsewhere [13]. In brief, this clinical cohort was previously examined and published using the behavioral and genetic data utilized in this study in a genome scan of schizophrenia [13]. As described previously [13] subjects were recruited from five geographic centers beginning in 1985: 213 families from the United States (based at Stony Brook, N.Y.), 50 from the United Kingdom (Oxford), 33 from Italy (Milan), 11 from Chile (Santiago), and two from Belgium (Leuven). Recruitment included catchment area screening, recruitment by health professionals at hospital and outpatient facilities, and advertisement through organizations which support families of mentally ill individuals. Written consent was obtained from all participants in the study after receiving an explanation of the study procedures and their implications. Consent was obtained using the same procedures in all five countries, and each center was granted approval with Single Project Assurance status by the Office of Protection From Research Risks of the U.S. Department of Health and Human Services. This study was conducted according to the principles expressed in the Declaration of Helsinki. Individual Institutional Review Board approval from the institutions to which the authors are affiliated were not obtained as this was a reanalysis of a previously published de-identified dataset.

### Samples and Subjects

#### Simulated Data (GAW14)

**Figure 1:**
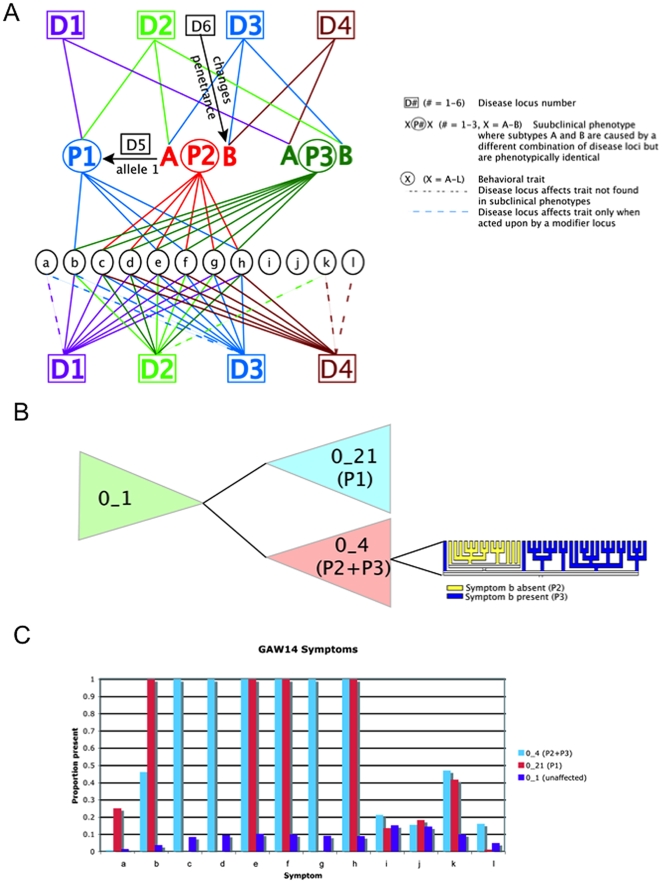
Genetic model and analysis of the GAW14 dataset. (**A**) Graphical representation of the genetic model used in the GAW14 simulation. D1-to-D4 are the major disease-causing loci while D5 and D6 are modifier loci that influence disease penetrance if the disease genotype is present. P1-to-P3 are latent phenotypes and a-to-l are sub-clinical phenotypes both caused by the disease loci, as seen by the connecting lines. The letters “A” and “B” associated with the latent phenotype reflect identical phenotypes, which are caused by different underlying loci or acted upon by modifier loci. (**B**) Graphical representation of the network showing relationships of behavioral phenotypes in the GAW14 dataset using a majority rule consensus tree. There are three key groupings referred to as clades 0_21, 0_4, and 0_1, which define the phenotypic groups P1, P2 and P3, and the unaffecteds respectively. The group shaded in blue, labeled 0_21 (P1), denotes the clade containing individuals who have the latent phenotype P1. The group in red labeled 0_4 (P2+P3) is defined in a similar manner. Finally, the group in green labeled 0_1 represents the unaffected individuals. The high level ratchet based tree search employed in the network generation procedure will not always resolve subtle differences within clades. Given that half of the individuals in clade 0_4 share certain traits, additional analyses within this clade were conducted to further refine the latent phenotypes. The clade 0_4 depicts the majority rule consensus tree resulting from a tree search within this clade. The expansion of clade 0_4 demonstrates the improved resolution of this clade as well as the separation of the latent phenotypes by the presence of trait b in P3 and the absence of trait b in P2. Specifically, the clade labeled in yellow is defined by the absence of trait b defining phenotype P2 and the clade labeled in blue have symptom b present defining phenotype P3. (**C**) A histogram plot for all sub-clinical traits present in the primary clades defined in part B. The x-axis represents the traits, each with three bars for each clade, and the y-axis represents the proportions of individuals with the presence of the trait. Within these clades, the proportions of individuals with each of the behavioral traits were examined and found to concur with the simulated models relating the behavioral traits with the latent phenotypes. In clade 0_21, 100% of individuals in this group have the traits b, e, f, and h, which define the P1 latent phenotype. The clade 0_4 is not as clearly resolved, where 100% of individuals share the behavioral traits c-to-h, but only fewer than 50% possess the traits b and k. This is because clade 0_4 defines the latent phenotypes P2 and P3 that are separated by only one behavioral trait (b) and share 6 traits in common (c-to-h). Finally, clade 0_1 contains most of the unaffected individuals not in clades 0_4 or 0_21 and is not defined by any one specific or subset of behavioral trait(s).

The GAW14 data was designed to model the genetic influences on a complex psychiatric disorder [14]. The made-up disorder termed Kofendred Personality Disorder (KPD) could be subcategorized into three distinct latent phenotypes denoted as P1, P2, and P3. These latent phenotypes were in turn defined by 12 behavioral traits labeled *a*-to-*l*. The genetic architecture of this disorder involves four disease gene loci D1, D2, D3, and D4 (Figure 1A). The genes interact in an epistatic fashion together with two modifier genes, D5 and D6 (Figure 1B). For the purposes of evaluating our methodology we assumed that the latent phenotypes were unknown, instead all analyses were based directly on the 12 behavioral traits.

In each population the families were ascertained based on the following criterion assuming a family contains at least one latent phenotype in order to be considered for inclusion in the study: for the Aipotu population at least two family members must have either P1, P2 or P3; for the Karangar population at least two family members must have P2 or P3; for the Danacaa population at least two family members must have P1. In this way, 100 families were ascertained from each population, constituting a single replicate (Replicate #1 was used in this study). A total of 300 families were analyzed, consisting of 2,077 individuals of which 781 were affected with KPD and 1,296 were unaffected. All genotype data for the 10 simulated chromosomes were examined, with 416 microsatellite markers approximately 7.5 centimorgans (cM) apart.

#### Real Data (Schizophrenia)

Reviewing the findings from the genome scan, we identified those chromosomes with moderate to significant linkage evidence[13]. The schizophrenia study included 1,779 subjects with probands and their relatives, who were previously examined and assigned to one of the following major diagnostic categories: schizophrenia, major depression, depression not otherwise specified, schizoaffective disorder, bipolar disorder, schizotypal personality disorder, psychosis not otherwise specified, or unaffected according to DSM-IV criteria [13] (with some individuals having been assigned an unknown diagnosis). Families with at least two members with diagnosis of schizophrenia were included in the study. In addition, 178 behavioral symptoms, as part of a Lifetime Symptom Checklist, were scored from the combined structured interviews, family informant information and medical records [15] across 1,779 individuals including both patients and family members. From these 178 behavioral symptoms, 158 were utilized in this study comprised of 154 dichotomous traits and four traits with four states (absent and minimally, moderately and severely affected). We then focused our efforts in analyzing these chromosomes in an effort to identify subsets of behavioral symptoms that may strengthen previous linkage findings, which were strictly based on major clinical diagnoses. To this end, 91 microsatellite markers were identified for this analysis (39 markers on chromosome 2, 30 markers on chromosome 10 and 22 markers on chromosome 22).

### Algorithm for Behavioral and Genetic Network Analysis

The hierarchical behavioral network representing phenotypic relatedness is obtained through the use of a character-based analysis. This approach optimizes the change of behavioral traits, where a change is defined by the loss (1→0) or gain (0→1) of the presence of a behavioral trait as the network is traversed from one individual to another[16]. This scoring system allows for the separation of clinical symptoms within diagnostic categories and for overlap between diagnostic categories. The structure of this network is then utilized to create increasingly more inclusive or nested sets of individuals based on phenotypic similarity each of which is tested via traditional linkage and/or association analyses. As such, the nesting of individuals based on the structure of the behavioral network allows for the identification of genetic loci that have both local and global influences on clusters of symptoms. Each group of individuals, defined by the nesting group derived from the behavioral network structure, is then considered as a candidate behavioral endophenotype (or a candidate phenotypic model) that is adopted for subsequent genetic linkage or association testing. If significant evidence for linkage or association is detected for a particular behavioral endophenotype, the symptom profile of that group can then be examined to identify those symptoms shared by the majority of individuals. Since the behavioral network algorithm maximizes the traits that individuals close to one another on the network share, the majority of individuals in a group with significant linkage or association are likely to all share one or more behavioral traits in common. In this way, relationships between specific endophenotypes and genotypes may be established.

The character-based algorithm described here, which generates a behavioral network based on individual symptoms, accounts for phenotypic heterogeneity. The goal is to identify genetically meaningful phenotypic groups. Since an optimal arrangement of shared behavioral symptoms was used to build the phenotypic network, the symptoms defining a phenotypic group of interest can easily be determined. As phenotypic groups are analyzed at multiple, increasingly inclusive levels, genetic loci that show linkage or association within small or large groups of individuals with particular symptoms can be detected (denoted as local or global affects respectively). This provides a direct means of detecting genetic loci that can potentially modulate behavioral symptoms.

**Figure 2:**
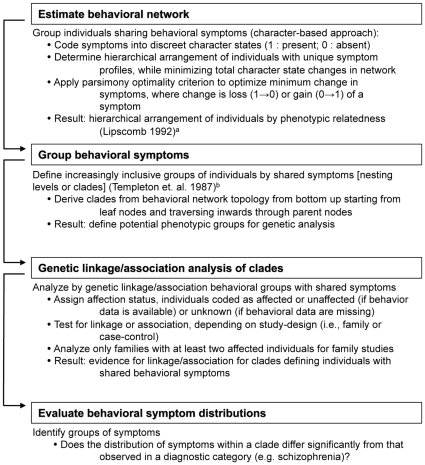
Pipeline of Algorithm for Behavioral and Genetic Analysis. ^a^ Templeton, A. R., Boerwinkle, E., Sing, C. F. 1987. A cladistic analysis of phenotypic associations with haplotypes inferred from restriction endonuclease mapping. I. Basic theory and an analysis of alcohol dehydrogenase activity in Drosophila. Genetics 117(2):343–351. ^b^ Lipscomb DL. 1992. Parsimony, homology and the analysis of multistate characters. Cladistics (8):45–65.

Specifically, our algorithm consists of four distinct components including (1) estimating a behavioral network, (2) nesting of the behavioral network into inclusive groups/clades[17], (3) performing genetic analysis, and (4) evaluating statistical significance of behavioral symptoms (see Figure 2 for an outline of the algorithm, and Figure S1 for an example of network estimation). The statistical evaluation of behavioral symptoms involves comparing symptom distributions of individuals belonging to a given nested group to the symptom distribution within the diagnostic category to which they were assigned. To this end, we adopt a likelihood-based approach to test for significant differences in the symptom distributions in these groups. The likelihood ratio test is formulated as follows,
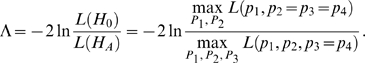



The likelihoods for the null and alternative hypotheses (H_0_ and H_A_ respectively) are straightforward, where 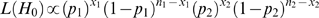
assuming *p_2_ = p_3_ = p_4_* with corresponding maximum likelihood estimates of 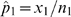; 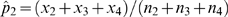. Similarly, 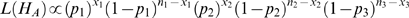 assuming *p_3_ = p_4_* with corresponding maximum likelihood estimates of 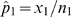; 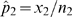 ; 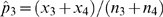. Note, *x_i_* is the number of individuals with symptoms in group *i* and *n_i_* is the total number of individuals in group *i*. Here *p* is the probability of the presence of a behavioral trait for any of four possible groupings, such that *p_2_* is the probability of observing a given trait in the clinical diagnostic group that does not overlap with the clade, and *p_3_* is that trait's probability in the clade of interest that does not overlap with the major diagnostic group. *p_4_* is the probability of trait overlap between *p_2_* and *p_3_*, and *p_1_* is the probability of the trait in individuals that are not in any of the previously noted groups. The null hypothesis (H_0_) assumes that the trait probability distribution for the individuals in the clade and the clinically defined diagnostic group (as well as the overlap) are the same (i.e., *p_2_ = p_3_ = p_4_*). Whereas, the alternative hypothesis (H_A_) assumes that the probability of the trait is not the same (i.e., *p_2_≠p_3_ = p_4_*). For the boundary conditions where one group is contained within the other, for H_A_ *p_2_* is compared to *p_4_*, while for H_0_ they are equal. The situation is similar in the case of no overlap between categories where *p_2_* and *p_3_* would be the key parameters. The likelihoods thus formulated are evaluated for each trait of interest and assessed via the likelihood ratio test at the parameter's maximum likelihood estimates. Furthermore, for assessment of statistical significance, empirical p-values were estimated using 10,000 permutations of the data.

### Data Analysis

Generating a network from the behavioral symptoms of individuals in the dataset involves examination of the entire “tree space”. This requires that all possible network configurations, which group individuals with respect to their shared symptom profiles must be enumerated and evaluated. This is not computationally feasible. Instead we used a heuristic ratchet search algorithm that has been developed for applications of exceptionally large datasets [18]. The ratchet search algorithm assumes that there are islands of local optimality in the search space for the shortest network [19], that is, a network with an arrangement of individuals and their shared symptoms that minimize the number of character state changes. However, such heuristic algorithms typically produce multiple equally parsimonious trees. A solution to this problem is to adopt a majority rule consensus tree, where a resolution among individual nodes on the tree is arrived at if it is supported by the majority of trees in the tree space. In this application, 51% is the minimum allowable support. For this analysis, all parsimony based ratchet searches were conducted using TNT v 1.0 [20]. All trees were visualized and all symptoms were mapped onto trees using MACCLADE v 4.08 [21]. To improve our tree resolution for a specific clade, we performed a tree search using the SPR branch swapping in PAUP v 4.0b10 [22].

The behavioral networks thus generated were examined and nested groups/clades were identified for genetic analysis. Since the datasets considered consist of family data, we performed genetic linkage analysis using the program MILINK from the LINKAGE software package [23], [24]. Heterogeneity LOD scores (HLOD) were computed based on an admixture likelihood model that jointly tests for linkage and heterogeneity (i.e., the maximum HLOD is compared with the log-likelihood under a null hypothesis of “no linkage and no heterogeneity” rather than with the maximum homogeneity LOD) [25], [26]. Linkage analyses were performed on those clades that were comprised of individuals from at least 40 families. This clade constraint was applied, *a priori*, before conducting the linkage analyses. This limited the number of tests performed for small samples with potentially little power to detect linkage. Of all nested groups considered, 6 clades met this criteria in the simulated dataset and 39 clades in the schizophrenia dataset. For each nested clade, LOD scores maximized over multiple models were calculated with corresponding estimated empirical p-values adjusting for the testing of multiple phenotypic and genotypic models as well as multiple marker loci. LOD scores were calculated under models of both homogeneity (LOD) and heterogeneity (HLOD)[26]. Four genetic models were considered including (1) a fully penetrant dominant model (Dom-1) and disease allele frequency of 0.01, (2) fully penetrant recessive model (Rec-1) and disease allele frequency of 0.09, (3) a dominant model with 55% penetrance (Dom-2) and phenocopy rate of 0.0005, and disease allele frequency of 0.01, and (4) a recessive model with 55% penetrance (Rec-2), phenocopy rate of 0.0005, and disease allele frequency of 0.09. The first two models (Dom-1 and Rec-1), though the simplest and perhaps overly optimistic, were chosen because they provide the greatest power to detect linkage [27]. The latter two models (Dom-2 and Rec-2) were based on a previously published analysis of this schizophrenia dataset[28]. In the absence of any knowledge regarding the mode of inheritance of the disease, as is typically the case for complex diseases, application of a limited set of simple genetic models have been shown to work well when testing for linkage [29].

To account for multiple testing, we estimated empirical p values, allowing for multiple phenotypic and genetic models, as well as multiple marker loci tested. To this end, we randomly assigned genotypes to the founders within each family while conditioning on the observed allele frequency and intermarker distances in the data and then mating individuals within families according to the pedigree structure using the program SIMULATE [30]. In this way, 100 randomized replicates of the data were generated. These replicates were used to estimate both the model-based and the global empirical p-values. The model-based p value (p_M_) is evaluated by recording the number of times a maximum LOD score from a replicate exceeds the maximum LOD score from that of the observed data, across all genetic models within each clade (phenotypic model) and therefore corrects for the testing of multiple genetic models and marker loci tested for a particular phenotypic model. The global p value (p_G_) is calculated by making comparisons to the maximum LOD scores from randomized replicates across all genotypic and phenotypic models (clades) and marker loci tested. This approach accounts for multiple testing of all combinations of genetic-phenotypic models as well as genetic markers tested.

## Results

### Simulated Data (GAW14)

Behavioral traits from all family members were analyzed to generate a behavioral network. The network correctly grouped the three latent phenotypes, as shown in Figure 1C. Within these groups, referred to as clades, the proportions of individuals with each of the behavioral traits were examined and found to concur with the simulated models relating the behavioral traits with the latent phenotypes (Figure 1C). Furthermore, genetic linkage analysis of these key clades identified the major disease loci contributing to KPD. In contrast to the findings observed from conducting a traditional genome scan (where KPD is used as the major diagnosis for defining affectedness), the observed linkage signals from this analysis were more significant (with the exception of one locus), and combinations of loci contributing to specific latent phenotypes were identified (Table S2). This analysis indicates that our algorithm is able to identify the genetic factors underlying each of the latent phenotypes. Indeed, this degree of resolution in detecting the contribution of genetic loci to specific sub-phenotypes in a complex trait model is not attainable using existing approaches.

### Real Data (Schizophrenia)

**Figure 3:**
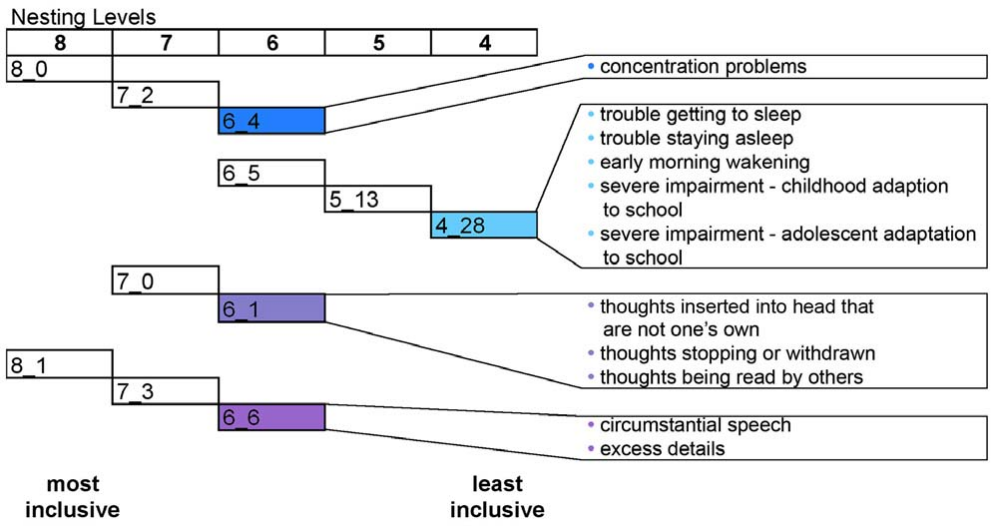
Nested structure of the network showing only the five clades that exhibit significant findings from the schizophrenia dataset. From left to right are the most to least inclusive nesting levels. The most inclusive nesting level, 8, divides the tree into three parts, clades 8_0, 8_1 and 8_2. The five clades found to have suggestive to significant linkage (4_28, 5_15, 6_1, 6_4, 6_6) are shaded with colors that are consistently used to represent them in the text. More inclusive nesting levels that encompass these five clades are also shown; however, nesting levels below level 8 that have no significant linkage findings are not shown. Clades with individuals who have a greater proportion of affective symptoms are grouped more closely together (4_28, 6_4). The clade with a greater proportion of positive symptoms typical of schizophrenia is grouped by itself (6_6), while the clade with both affective and positive symptoms is grouped between the two (6_1), and the clade with individuals with minimal symptoms is grouped by itself (5_15).

**Figure 4:**
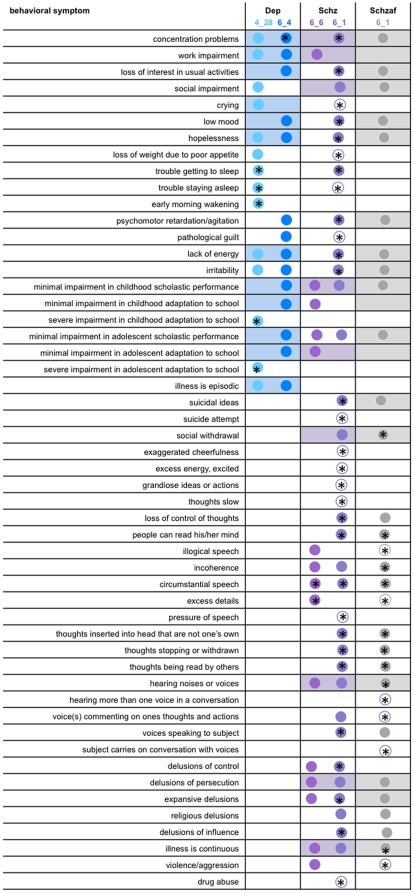
The spectrum of behavioral symptoms defining the depression and schizophrenia clades. Symptoms present in greater than 70% of individuals with the diagnosis of depression, schizophrenia, or schizoaffective disorder are depicted as filled-in circles with depression (in blue), schizophrenia (in purple) and schizoaffective (in gray). Significant differences in symptom distributions within clades compared to the diagnostic categories of depression (Dep), schizophrenia (Schiz), and schizoaffective (Schizaf) are depicted with a “*” (with statistical significance reported at the 0.05 level). Unfilled circles with a “*” imply statistically significant differences between the clade and diagnostic category but that less than 70% of individuals in that clade have that symptom. Boxes which encompass the circles in the three columns of depression, schizophrenia and schizoaffective disorder are shaded when that symptom appears in greater than 70% of the individuals in that diagnostic category. Five symptoms distinguish the *depression^4_28^* clade from the diagnostic category of depression, which include trouble getting to sleep, trouble staying asleep, early morning awakening, severe impairment in both childhood and adolescent adaptation to school. The *depression^6_4^* clade is distinguished by one symptom, mainly difficulty concentrating. These depression clades are mutually exclusive. Further, language disorder symptoms distinguish the *schizophrenia^6_6^* clade from the diagnostic category schizophrenia (i.e., circumstantial speech and excess details) as well as an increased incidence of violence and aggression. The schizophrenia^6_1^ clade is distinguished from both diagnostic categories schizophrenia and schizoaffective disorder by three symptoms, including thoughts inserted into one's head that are not one's own, thoughts stopping or withdrawn, and thoughts being read by others. However, this clade shares behavioral symptoms characteristic of both of these diagnostic categories.

Analysis of schizophrenia samples identified four distinct behavioral groups (clades) with corresponding evidence for genetic linkage. The salient clinical features of these groups were similar to major depression and schizophrenia with two groups resembling “*depression*” (clades 6_4 and 4_28) and two “*schizophrenia*” (clades 6_6 and 6_1). These clades can be represented in a behavioral network (Figure 3). In this network representation, the most inclusive nesting level contains two clades (8_0 and 8_1) that encompass the four clades noted above, where these clades become smaller and less inclusive as the network is traversed from left to right (Figure 3). In this way, individuals with shared behavioral symptoms are grouped through successive nesting levels, in that specific symptom(s) are shown to define specific clades (Figure 3). The significance of these specific symptom profiles was further evaluated (Figure 4). Those behavioral symptoms that were observed in >70% of the individuals within each clade were identified, and the relative proportion of these symptoms were compared to the assigned diagnostic categories. Specifically, those symptoms that occur in a significantly greater number of individuals in the clade under consideration were compared to the diagnostic category (of e.g., depression or schizophrenia) to which the majority of the individuals in the clade were assigned. In this way, statistically significant symptom profiles were identified that characterize each clade (Figure 4).

The depression group *depression* ^6_4^ is characterized by the symptom “difficulty concentrating” (Figure 4), since the majority of individuals belonging to this clade share this symptom. In contrast, the second clade, *depression* ^4_28^ is characterized by a spectrum of symptoms, that include “trouble getting to sleep”, “trouble staying asleep” and “early morning wakening”, and “severely poor adaptation to school with poor scholastic performance”. Such symptoms appear in significantly greater proportions of individuals in these clades than those with the assigned diagnosis of major depression (Figure 4). Further, these individuals belong to mutually exclusive groups accounting for 83 of the 139 patients with major depression in the total dataset (Table 1), identifying two depression sub-phenotypes with distinct symptom profiles.

**Table 1:** Diagnostic and LOD score statistics for clades.

	Depression^6_4^	Depression^4_28^	Schizophrenia^6_6^	Schizophrenia^6_1^
**schizophrenia**	4	0	56	56
**schizoaffective**	9	0	2	32
**major depression**	40	43	0	0
**depression NOS**	9	5	1	0
**bipolar**	11	0	0	2
**schizotypal PD**	4	3	1	0
**psychosis NOS**	0	1	2	2
**normal**	6	5	0	0
**unknown**	17	7	7	8
**number families**	64	46	52	77
**number individuals**	100	64	69	100
**Z_max_ (α)**	4.07 (0.86)	2.86 (0.50)	2.19 (1)	2.42 (0.68)
**P_M_**	≤0.01	0.02	0.06	0.02
**P_G_**	0.08	0.6	0.79	0.94
**marker**	D2S2248	D2S160	D2S168	D2S2298
**model**	Rec-1	Rec-1	Dom-2	Rec-2

For the four significant depression and schizophrenia clades, the number of individuals from each diagnostic group that are present in each clade and corresponding LOD scores are reported. The clades are separated into two groups, consisting of individuals with symptoms typical of depression and those with individuals with symptoms typical of schizophrenia. The maximum LOD score is reported for each clade, maximized over all genetic models and analysis schemes examined. The term HLOD denotes the maximum heterogeneity LOD score and α is the corresponding heterogeneity parameter. Model refers to the genetic model as described in the text. P_M_ and P_G_ are the estimated model-based and global empirical p values respectively.

Linkage analysis of these depression clades provided significant evidence for linkage on chromosome 2 (Table 1). Individuals with behavioral symptoms in each of the depression groups were designated as affected and their family members who did not share these defining symptoms were designated as unaffected for consideration in the linkage analyses. We observed significant linkage signal for the *depression^6_4^* clade with loss of concentration as the defining symptom on chromosome 2q35 (heterogeneity LOD score HLOD = 4.07 at marker D2S2248). We also observed suggestive evidence for linkage on 2q13 for the second *depression^4_28^* clade (HLOD of 2.86), where the dominating symptoms were those of sleep disturbances. Comparatively, analysis of families in the total dataset using the diagnosis of major depression to define affectedness yielded lower linkage signals. In fact, the maximal linkage signal overlapped with the same chromosomal region identified by the *depression^4_28^* clade, with a lower score (HLOD = 2.6 with 'alpha = 1 at marker D2S160; Figure S2). The next highest linkage signal was 5 centiMorgans (cM) away from our strongest finding with the *depression^6_4^* clade with a substantially lower LOD score (HLOD = 2.1 and alpha = 0.45 at marker D2S126; Figure S2). Taken together, these results may indicate the presence of two distinct candidate loci influencing the symptoms defining these two groups, since the clades contain two mutually exclusive groups of individuals with potentially stronger, more homogeneous signal resulting from such refinement of phenotypes.

The schizophrenia groups also revealed intriguing symptom profiles. The *schizophrenia^6_6^* clade was characterized by language disorder symptoms such as circumstantial speech and excess details, and aggressive and violent tendencies (Figure 4). The *schizophrenia^6_1^* clade was composed mainly of individuals with the major diagnoses of schizophrenia or schizoaffective disorder with symptoms including loss of concentration, pressure of speech, low mood, suicidal ideation and psychomotor retardation (Figure 4). Individuals belonging to this clade have symptoms more characteristic of those with the diagnosis of schizoaffective disorder even though the majority of these individuals had the diagnosis of schizophrenia (56% schizophrenia vs. 32% schizoaffective). This group is enriched with language disorder and hallucination symptoms resembling schizophrenia and depressive symptoms resembling schizoaffective disorder. This is a clear demonstration of the overlap between and difficulty in separating these two so called clinically distinct diagnostic categories.

Linkage analysis of these schizophrenia clades provided suggestive evidence for linkage (Table 1). We observed a novel linkage finding with *schizophrenia* ^6_6^ clade on chromosome 2p25 (HLOD of 2.19 and alpha  = 0.66 proximal to marker D2S168). This linkage peak is approximately 66 cM from the peak LOD score of 2.99 reported previously [13] from linkage analysis of the same samples with the diagnosis of schizophrenia or schizoaffective disorder. Linkage analysis with the schizophrenia only diagnosis resulted in substantial improvement of the LOD score rising to 5.13 in the same region when only those with schizophrenia were considered as affected [31]. The analysis of the *schizophrenia^6_1^* clade yielded maximal linkage signal on chromosome 2p21 (HLOD of 2.42 and alpha  = 1 at marker D2S2298; no evidence for linkage was observed at this locus in the comparative analysis with diagnosis of schizophrenia or schizoaffective disorder for affectedness (Table 1). These findings are unique to each schizophrenia clade with a relative distance of 19 cM between the most significant loci from each clade, underscoring our ability to detect potential linkage to distinct genetic regions due to the refinement in the phenotypic definitions.

Finally and most importantly, the present study also illustrates that behavioral symptoms follow a continuum of psychosis and extend into normal personality traits. This is best illustrated by clade 5_15, which has no defining symptom profile and encompasses a collection of individuals from many diagnostic categories with the largest represented group being the unaffecteds. Many of these individuals have very few symptoms, which is why they cluster more closely with the unaffected individuals. However, closer examination of their symptom distribution revealed that approximately 57% of all individuals carry at least one symptom of a personality disorder (data not shown), including paranoid, schizoid, schizotypal and borderline traits (with significant evidence for linkage, HLOD of 3.4 proximal to D2S391 approximately 4 cM from the peak LOD in schizophrenia^6_1^ clade see Table S1). This result captures the notion of a continuum of psychosis. As expected, unaffected family members of the mentally ill often share some symptoms with their affected family members.

## Discussion

In this study, we have developed an analytical framework for the characterization of behavioral symptoms with shared genetic contribution. This method is a departure from the traditional approaches to analysis of neuropsychiatric disorders in that it does not rely on *a priori* assignment of individuals to specific diagnostic categories. We have demonstrated the effectiveness of this approach in identifying the genetic factors involved in the etiology of complex phenotypic models using simulated data. Application of this method to our schizophrenia data revealed groupings of patients whose characteristic symptoms are novel with potential involvement of gene(s) contributing to these symptoms. With this method, we further refined previous linkage findings in this dataset, providing improvements in the linkage results by limiting potential phenotypic heterogeneity. Analysis of individuals with shared behavioral symptoms is a powerful and straightforward approach to identify the genetic factors underlying such symptom profiles and will lead to discovery of endophenotypes that are likely to be more biologically meaningful than standard diagnostic categories.

Our results clearly demonstrate the separation of individuals with major depression on the basis of whether they exhibit sleep disturbance symptoms. These physiological symptoms may distinguish the etiological factors for the disorder in each group. Further, the separation of the two linkage peaks for major depression on chromosome 2 based on two primary behavioral symptoms (i.e., sleep disturbances and difficulty concentrating) may indicate the identification of two groups of patients whose symptoms have two distinctly different genetic origins. The individuals in the group suffering from sleep disturbances appear to have more severe symptoms during childhood, indicating a possible prodrome or an earlier age of onset. This increases the likelihood that these symptoms may have high genetic loading, consistent with epidemiological studies, suggesting a strong correlation between the diagnosis of depression and sleep disturbances [32], [33], [34]. The second depression group is comprised of individuals that have difficulty concentrating, confirming previous findings [35], [36], [37]. This group provided the strongest linkage evidence in our study, where the linkage signal spans the genomic region containing the gene cAMP responsive element binding protein 1 (CREB1) which has been implicated as a candidate gene for depression [38], [39].

The most striking schizophrenia finding involved the group of individuals with language disorders, considered central to the symptom pathology of schizophrenia [40], [41]. It has been suggested that these symptoms are related to cognitive deficits in schizophrenia [42]. We observed one group with over 95% of individuals defined by specific symptoms of language disorders, such as circumstantial speech and excess details. These could represent a core set of symptoms for a specific subtype of schizophrenia with a distinct genetic pathway that might be targeted with specific pharmacological and cognitive treatments. While there have been numerous studies reporting linkage to schizophrenia on chromosome 2 [31], [43], [44], [45], [46], [47], [48], [49], [50] none have specifically reported linkage at the sites identified in the analysis of our two subgroups of schizophrenia patients at chromosomal regions 2p25.1 and 2p21. The region 2p25.1 contains the gene neurotensin receptor 2, which has an important physiological role in sensory perception [51]. Additionally, the region 2p21 contains the gene protein kinase C epsilon, which has been shown to be involved in neuronal channel activation and may be involved in emotional learning and memory [52]. These results demonstrate the efficacy of our approach, however they are subject to replication in future studies using the wealth of genetic and behavioral data collected in other linkage and association studies.

While we grouped individuals based primarily on shared behavioral symptoms, it is also possible to include physiological trait measurements, as well as non-genetic or environmental factors. In future analyses, the relative importance of traits can be weighted when building the network based on the heritability of the trait. Traits with high heritability would contribute more to the structure of the network defining phenotypic relationships. The analytical procedure is also easily extendable to association studies using a case-control study design by simply performing association tests on phenotypic groups instead of linkage analyses. This method is ideally suited for application to diseases where clinical heterogeneity of phenotypes is suspected and multiple symptoms are recorded for study subjects, as is typically the case for most neuropsychiatric disorders.

Our study shows that reconsideration and refinement of phenotypic definitions will reveal a myriad of new phenotypic and genotypic relationships. Although the representation of behavioral symptoms among individuals is not likely to be hierarchical, with some genes affecting multiple symptoms and multiple genes having an effect on an individual symptom, our method aims to generate hypotheses regarding specific subgroups of individuals with shared symptoms and to delineate the genetic basis of such behavioral symptoms. In this way, individuals with shared symptom profiles will be grouped together and genes with effects on these symptoms will be detected, generating novel phenotype-genotype relationships.

## Supporting Information

Figure S1 Simplified example of a network representing relationships between patient's behavioral phenotypes. This shows how behavioral symptoms of individuals in the dataset are used to group patients together into a hierarchical network. (A) Depiction of eight patients each scored for the presence (1) or absence (0) of ten behavioral symptoms. (B) Network representing the grouping of patients based on phenotypic relatedness. Patients who share more behavioral symptoms in common are grouped together, where the closest neighbors in a network are binned together into a more inclusive group (referred to as nesting level).
(8.21 MB TIF)

Figure S2 Depression LOD score plots. This demonstrates the separation and amplification of linkage peaks in the two mutually exclusive depression groups as compared to the overall diagnoses of depression. The LOD score peak at approximately 122 cM circled for the depression diagnoses (panel A) is significantly amplified in the depression4_28 clade (panel B), which also lacks a LOD score peak at ∼218 cM position. In contrast, the depression6_4 clade shows amplification of the peak at ∼218 cM (panel C), corresponding to the second peak for the depression diagnoses (panel A). Interestingly, the depression6_4 clade lacks the peak at approximately 122 cM, present in the depression4_28 clade (panels B and C). These two depression clades are mutually exclusive, indicating a potential separation of two genetically distinct groups of individuals within the diagnostic category major depression who can be distinguished phenotypically by the presence/absence of sleep disturbance symptoms.
(9.47 MB TIF)

Table S1 Diagnostic and LOD score statistics for clade 5_15. For this clade, the number of individuals carrying each diagnosis is provided as well as the number of individuals and families and the relevant linkage statistics. The maximum LOD score is reported maximized over all genetic models and analysis schemes examined with corresponding model parameters. The term HLOD denotes the maximum heterogeneity LOD score and α is the corresponding heterogeneity parameter. Marker refers to the genetic marker with the observed maximum HLOD. Model refers to the genetic model as described in the text. PM and PG are the estimated model-based and global empirical p-values respectively.
(0.04 MB DOC)

Table S2 Linkage Results of Simulated GAW14 Data. Clade refers to the clades under consideration, Phenotype refers to the latent phenotypes, HLOD is the maximum heterogeneity LOD score and α is the heterogeneity parameter, Model refers to the genetic model as described in the text, and PM and PG are the estimated model-based and global empirical p-values respectively. Chromosome is the chromosome on which this peak occurs, position is the position on the chromosome at which the peak occurs in cM, and disease locus is the disease locus that it identifies. Details of the genetic models and analyses as well as the empirical p-value estimations are given in main text. (A) Linkage analysis of the clade (0_21) containing the latent phenotype P1 showed strong linkage to the disease gene D1 on chromosome 1, which together with D2 define the underlying genetic contribution for P1. Analysis of clade 0_4 also revealed significant evidence for linkage with the disease genes D3 and D4 contributing to the latent phenotypes P2 and P3. However, the disease locus D2 was not detected in linkage analysis of these clades, which included individuals who harbored the latent phenotypes of interest. This is likely due to the fact that the latent phenotypes P2 and P3 are caused by alternate epistatic interactions between the disease loci D2 and D3, where for P2 the disease loci D2 has a recessive mode of inheritance and D3 has a dominant mode of inheritance, and in contrast for P3 the disease loci D2 has a dominant mode of inheritance and D3 has a recessive mode of inheritance. As for the linkage analysis there was no resolution between the phenotypic groups P2 and P3. Thus, they were analyzed with these opposite modes of inheritance which coupled with the reduced sample size resulting from subdividing this group reduced the potential genetic signal for the disease loci D2. Interestingly, D2 was correctly localized when the clade containing the majority of the unaffecteds was examined. The disease locus D2 is the genetic determinant for traits e, f and h in unaffected individuals, which acts in a dominant manner with a penetrance of approximately 20%. (B) Whole-genome scan analysis on the entire dataset using Kofendred Personality Disorder as the major diagnosis (i.e., used to define affectedness status). All the major disease loci were also identified in this analysis, yet with less significant linkage signals. The exception was locus D2 which had a more significant HLOD score in this genome scan. (C) The two clades separated by the presence (P3) or absence (P2) of trait b were examined via linkage analysis, and loci D1, D2 and D4 were successfully isolated within the separated phenotypic groups P2 and P3.
(0.05 MB DOC)
