# Supplementary figures and images for: A Novel Analytical Framework for Dissecting the Genetic Architecture of Behavioral Symptoms in Neuropsychiatric Disorders
Source: PLoS One. 2010 Mar 16;5(3):e9714. doi: 10.1371/journal.pone.0009714 (PMC2838792; doi:10.1371/journal.pone.0009714)

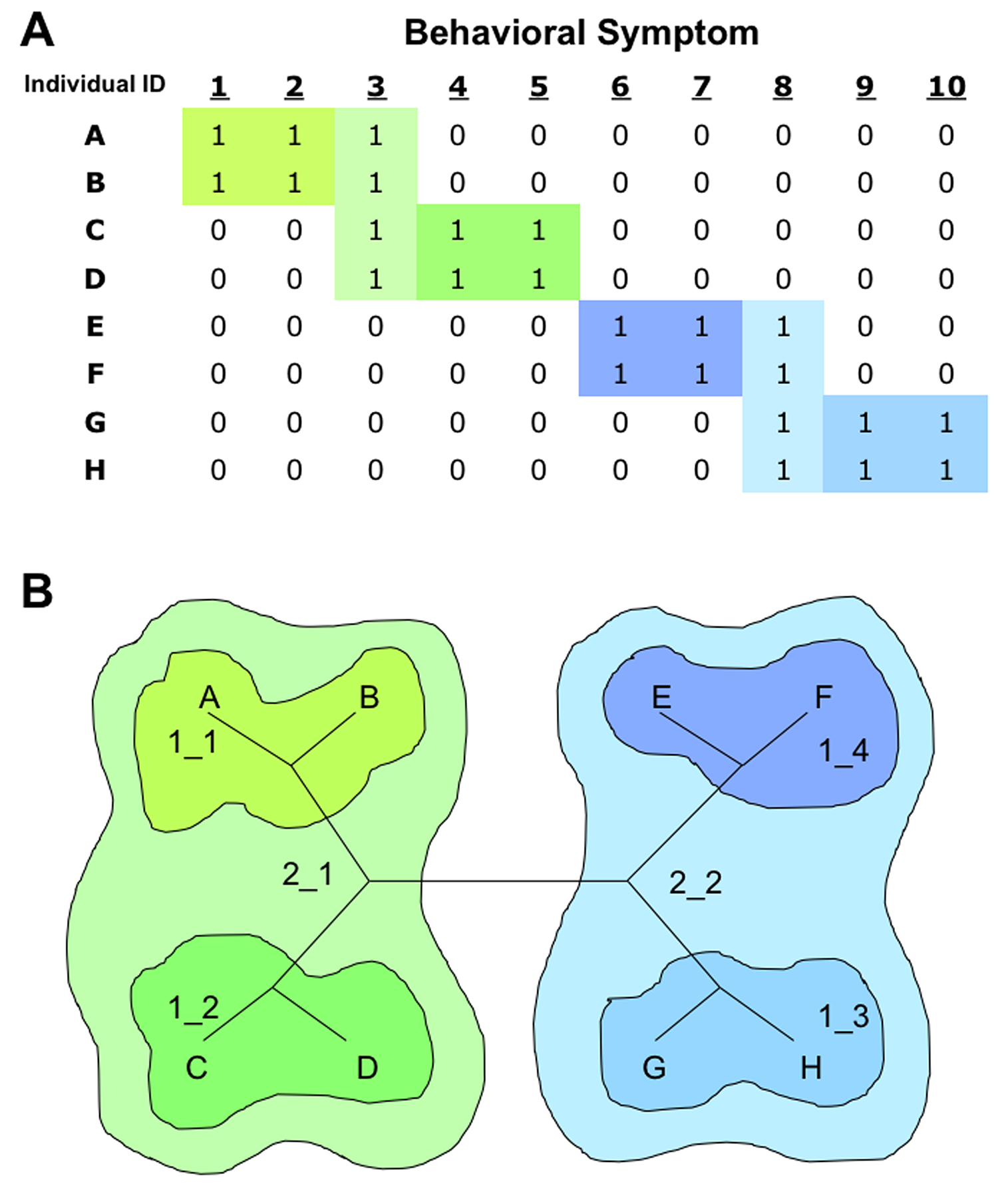

Supplement: Figure S1 — Simplified example of a network representing relationships between patient's behavioral phenotypes. This shows how behavioral symptoms of individuals in the dataset are used to group patients together into a hierarchical network. (A) Depiction of eight patients each scored for the presence (1) or absence (0) of ten behavioral symptoms. (B) Network representing the grouping of patients based on phenotypic relatedness. Patients who share more behavioral symptoms in common are grouped together, where the closest neighbors in a network are binned together into a more inclusive group (referred to as nesting level). (8.21 MB TIF) [file pone.0009714.s001.tif]

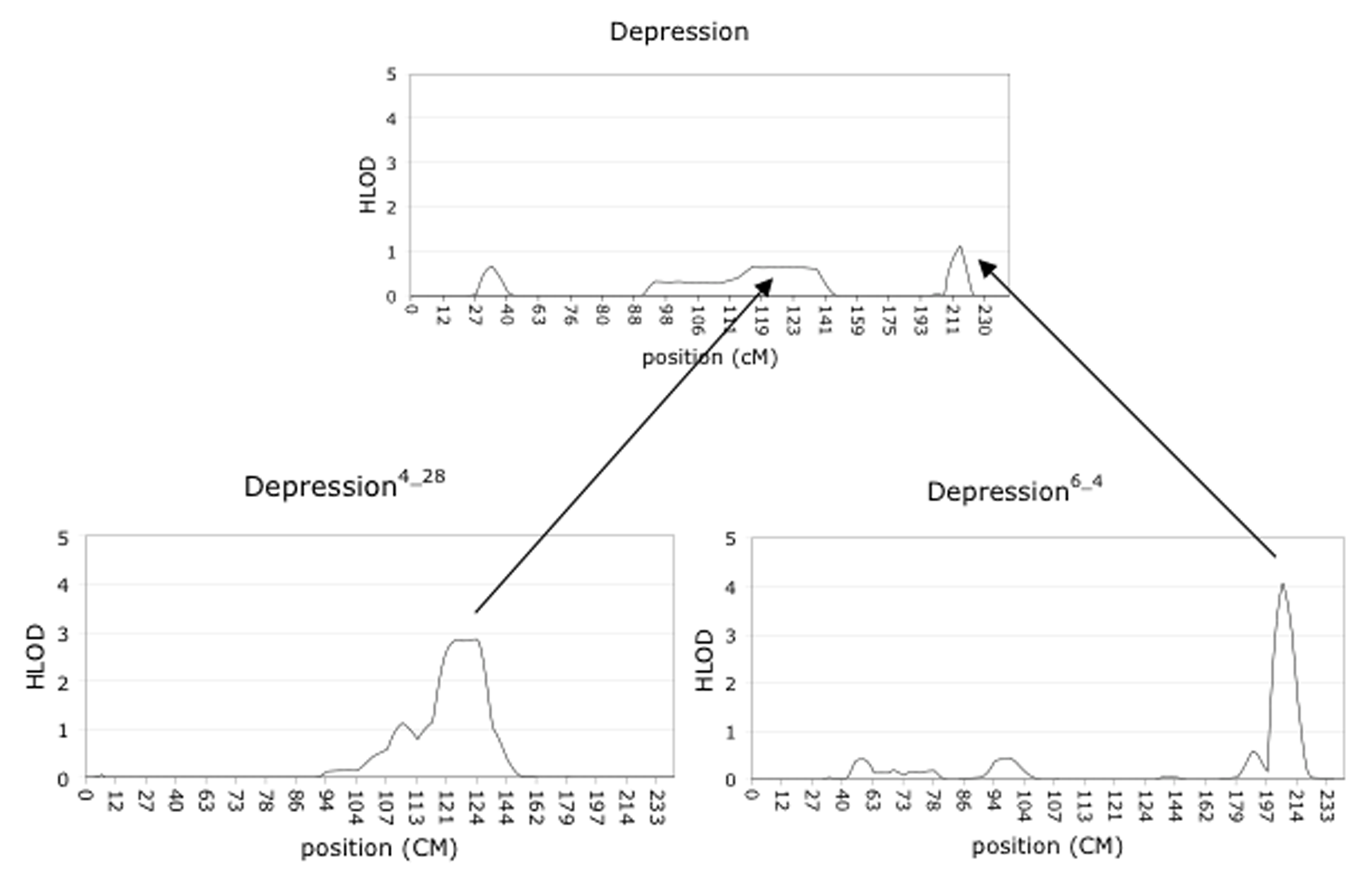

Supplement: Figure S2 — Depression LOD score plots. This demonstrates the separation and amplification of linkage peaks in the two mutually exclusive depression groups as compared to the overall diagnoses of depression. The LOD score peak at approximately 122 cM circled for the depression diagnoses (panel A) is significantly amplified in the depression4_28 clade (panel B), which also lacks a LOD score peak at ∼218 cM position. In contrast, the depression6_4 clade shows amplification of the peak at ∼218 cM (panel C), corresponding to the second peak for the depression diagnoses (panel A). Interestingly, the depression6_4 clade lacks the peak at approximately 122 cM, present in the depression4_28 clade (panels B and C). These two depression clades are mutually exclusive, indicating a potential separation of two genetically distinct groups of individuals within the diagnostic category major depression who can be distinguished phenotypically by the presence/absence of sleep disturbance symptoms. (9.47 MB TIF) [file pone.0009714.s002.tif]
