# Supplementary material for: A Novel Analytical Framework for Dissecting the Genetic Architecture of Behavioral Symptoms in Neuropsychiatric Disorders
Source: PLoS One. 2010 Mar 16;5(3):e9714. doi: 10.1371/journal.pone.0009714 (PMC2838792; doi:10.1371/journal.pone.0009714)
Supplement: Table S1 — Diagnostic and LOD score statistics for clade 5_15. For this clade, the number of individuals carrying each diagnosis is provided as well as the number of individuals and families and the relevant linkage statistics. The maximum LOD score is reported maximized over all genetic models and analysis schemes examined with corresponding model parameters. The term HLOD denotes the maximum heterogeneity LOD score and α is the corresponding heterogeneity parameter. Marker refers to the genetic marker with the observed maximum HLOD. Model refers to the genetic model as described in the text. PM and PG are the estimated model-based and global empirical p-values respectively. (0.04 MB DOC) [file pone.0009714.s003.doc]

|  | **Clade 5_15** |
| --- | --- |
| **schizophrenia** | 33 |
| **schizoaffective** | 2 |
| **major depression** | 8 |
| **depression NOS** | 29 |
| **bipolar** | 2 |
| **schizotypal PD** | 14 |
| **psychosis NOS** | 1 |
| **normal** | 43 |
| **unknown** | 29 |
| **number families** | 96 |
| **number individuals** | 161 |
| **Z_max_ (α)** | 3.35 (0.56) |
| **P_M_** | ≤0.01 |
| **P_G_** | 0.27 |
| **marker** | D2S391 |
| **model** | Rec-1 |
