# Supplementary material for: A Novel Analytical Framework for Dissecting the Genetic Architecture of Behavioral Symptoms in Neuropsychiatric Disorders
Source: PLoS One. 2010 Mar 16;5(3):e9714. doi: 10.1371/journal.pone.0009714 (PMC2838792; doi:10.1371/journal.pone.0009714)
Supplement: Table S2 — Linkage Results of Simulated GAW14 Data. Clade refers to the clades under consideration, Phenotype refers to the latent phenotypes, HLOD is the maximum heterogeneity LOD score and α is the heterogeneity parameter, Model refers to the genetic model as described in the text, and PM and PG are the estimated model-based and global empirical p-values respectively. Chromosome is the chromosome on which this peak occurs, position is the position on the chromosome at which the peak occurs in cM, and disease locus is the disease locus that it identifies. Details of the genetic models and analyses as well as the empirical p-value estimations are given in main text. (A) Linkage analysis of the clade (0_21) containing the latent phenotype P1 showed strong linkage to the disease gene D1 on chromosome 1, which together with D2 define the underlying genetic contribution for P1. Analysis of clade 0_4 also revealed significant evidence for linkage with the disease genes D3 and D4 contributing to the latent phenotypes P2 and P3. However, the disease locus D2 was not detected in linkage analysis of these clades, which included individuals who harbored the latent phenotypes of interest. This is likely due to the fact that the latent phenotypes P2 and P3 are caused by alternate epistatic interactions between the disease loci D2 and D3, where for P2 the disease loci D2 has a recessive mode of inheritance and D3 has a dominant mode of inheritance, and in contrast for P3 the disease loci D2 has a dominant mode of inheritance and D3 has a recessive mode of inheritance. As for the linkage analysis there was no resolution between the phenotypic groups P2 and P3. Thus, they were analyzed with these opposite modes of inheritance which coupled with the reduced sample size resulting from subdividing this group reduced the potential genetic signal for the disease loci D2. Interestingly, D2 was correctly localized when the clade containing the majority of the unaffecteds was examined. The disease l [file pone.0009714.s004.doc]

| **A** |  |  |  |  |
| --- | --- | --- | --- | --- |
| **clade** | **0_21** | **0_4** | **0_4** | **0_1** |
| **phenotype** | P1 | P2, P3 | P2, P3 | Unaffected |
| **Z_max_ (α)** | 7.516 (0.55) | 8.472 (0.32) | 3.448 (0.30) | 6.695 (0.12) |
| **p_M_** | ≤0.01 | ≤0.01 | 0.03 | ≤0.01 |
| **p_G_** | ≤0.01 | ≤0.01 | 0.3 | ≤0.01 |
| **chromosome** | 1 | 9 | 5 | 3 |
| **position (CM)** | 158.21 | 1.51 | 2.92 | 291.35 |
| **disease locus** | D1 | D4 | D3 | D2 |
| **model** | Dom-2 | Rec-2 | Dom-2 | Dom-1 |

| **B** |  |  |  |  |
| --- | --- | --- | --- | --- |
| **disease locus** | **D1** | **D2** | **D3** | **D4** |
| **Z_max_ (α)** | 7.45 (0.32) | 9.64 (0.35) | 3.18 (0.11) | 5.52 (0.17) |
| **chromosome** | 1 | 3 | 5 | 9 |
| **position (CM)** | 171.77 | 313.94 | 0 | 0 |
| **model** | Dom-2 | Dom-2 | Rec-2 | Rec-2 |

| **C** |  |  |  |  |
| --- | --- | --- | --- | --- |
| **clade** | **Blue** | **Blue** | **Yellow** | **Yellow** |
| **phenotype** | P3 | P3 | P2 | P2 |
| **Z_max_ (α)** | 2.921 (0.46) | 4.06 (0.32) | 4.864 (0.31) | 2.773 (0.16) |
| **p_M_** | 0.04 | ≤0.01 | ≤0.01 | 0.04 |
| **p_G_** | 0.31 | 0.02 | ≤0.01 | 0.46 |
| **chromosome** | 1 | 9 | 3 | 9 |
| **position (CM)** | D01S0021 | D09S0347 | D03S0126 | D09S0347 |
| **disease locus** | D1 | D4 | D2 | D4 |
| **model** | Dom-2 | Rec-2 | Rec-2 | Rec-1 |
